# Immune‐related dermatitis during combined treatment with pembrolizumab and axitinib in a patient with metastatic renal cell\x92carcinoma with stasis dermatitis

**DOI:** 10.1002/iju5.12356

**Published:** 2021-08-05

**Authors:** Shunsuke Imai, Masaki Nakamura, Satomi Chujo, Ryousuke Ooki, Yasushi Inoue, Hajime Horiuchi, Teppei Morikawa, Keita Uchino, Atsuyuki Igarashi, Yoshiyuki Shiga

**Affiliations:** ^1^ Urology NTT Medical Center Tokyo Tokyo Japan; ^2^ Department of Dermatology NTT Medical Center Tokyo Tokyo Japan; ^3^ Department of Medical Oncology NTT Medical Center Tokyo Tokyo Japan; ^4^ Department of Diagnostic pathology NTT Medical Center Tokyo Tokyo Japan

**Keywords:** axitinib, immune‐related adverse event, pembrolizumab, renal cell carcinoma, stasis dermatitis

## Abstract

**Introduction:**

The combination of pembrolizumab and axitinib has recently been approved as a first‐line treatment for previously untreated metastatic renal cell carcinoma. However, immune‐related adverse events are not well known.

**Case presentation:**

A 65‐year‐old male was diagnosed with renal cell carcinoma with metastases to the brain and lungs. The patient had a medical history of stasis dermatitis. During the combined treatment of pembrolizumab and axitinib, blisters appeared on the lower extremities. Skin biopsy revealed septal panniculitis, pustules, and perivascular lymphocytic and neutrophilic infiltration of the skin, and the patient was diagnosed with immune‐related dermatitis. The dermatitis improved with oral prednisolone treatment.

**Conclusion:**

A case of immune‐related dermatitis during combinatorial treatment with pembrolizumab and axitinib for renal cell carcinoma has been reported. Preexisting stasis dermatitis may have affected the onset and deterioration of immune‐related dermatitis.

**Abbreviations**
CT: computed tomography
NA: not analyzed
PD‐1: programmed cell death protein 1
PD‐L1: programmed cell death ligand 1
RCC: renal cell carcinoma
TKIs: tyrosine kinase inhibitors

**Keynote message**

Immune‐related adverse events associated with combined pembrolizumab and axitinib treatment are not well known. We report a case of immune‐related dermatitis in a patient with metastatic renal cell carcinoma and preexisting stasis dermatitis. Diverse pathological findings from skin biopsy and the progression of clinical findings led to a diagnosis of immune‐related dermatitis.

## Introduction

Antiangiogenic drugs are currently the main treatment option for metastatic RCC. RCC is characterized by a highly angiogenic microenvironment; therefore, several TKIs have been approved for the treatment of patients with metastatic RCC.^1^ Axitinib, a TKI that blocks VEGF receptors 1, 2, and 3, was approved as a second‐line drug for metastatic or advanced RCC.^2^

Pembrolizumab is a monoclonal antibody against PD‐1 that inhibits the interaction between PD‐1 and its ligand, PD‐L1. PD‐1 is expressed on the surface of activated T cells, natural killer cells, and B cells, while PD‐L1 is expressed on tumor cells,^3^ which are known to suppress T‐cell proliferation and antitumor responses through PD‐1/PD‐L1 interaction. This blockade results in sustained T‐cell activation and antitumor activity.^4^

Recently, combination treatment with antiangiogenic agents and immune checkpoint inhibitors, including pembrolizumab, has become a promising treatment option for metastatic RCC. The combination of pembrolizumab and axitinib is a newly approved treatment for previously untreated advanced RCC.^5^ This combination treatment showed improved anticancer effects over sunitinib, which is currently one of the main treatment options for advanced RCC among other immune‐oncology drugs.^6^, ^7^

Dermatitis is one of the most common immune‐related adverse events (irAEs) in patients with advanced RCC undergoing pembrolizumab treatment.^8^ Although most cutaneous irAEs are mild and less than 1%–3% progress to grade 3 or 4 disease in anti‐PD‐1‐treated patients,^5^, ^9^, ^10^ severe dermatitis could result in treatment discontinuation in some patients. Herein, we report a case of immune‐related severe dermatitis that occurred in a patient with metastatic RCC treated with a combination of pembrolizumab and axitinib.

## Case

A 65‐year‐old male was referred to our hospital with a suspected metastatic brain tumor on head magnetic resonance imaging and difficulty in mobilizing the right upper limb. CT revealed a right renal mass and multiple lung metastases. The patient presented with stasis dermatitis and had a 45‐year history of smoking (20–40 cigarettes a day; Brinkman Index, 900–1800). Stasis dermatitis was considered to be due to compression of inferior vena cava by the renal mass, and treatment with clobetasol propionate have started.

Following gamma knife treatment for the metastatic brain tumor of the left frontal lobe, CT‐guided needle biopsy of the renal tumor was performed. Histopathological examination revealed clear cell RCC of the right kidney.

**Fig. 1:**
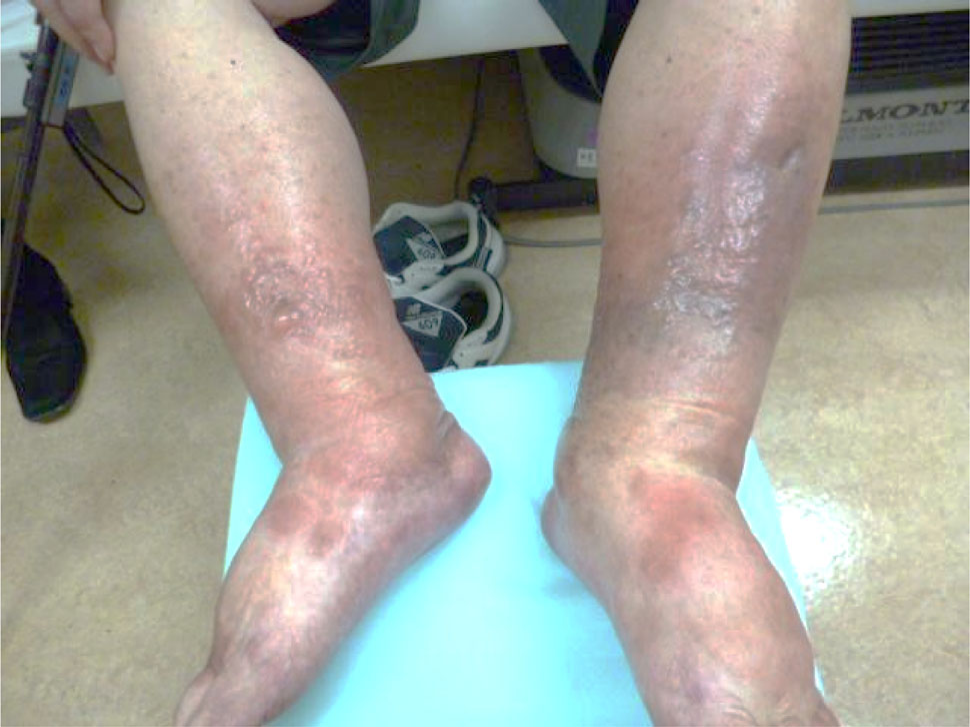
Representative picture of edema of the lower extremities after a single dose of pembrolizumab and axitinib.

**Fig. 2:**
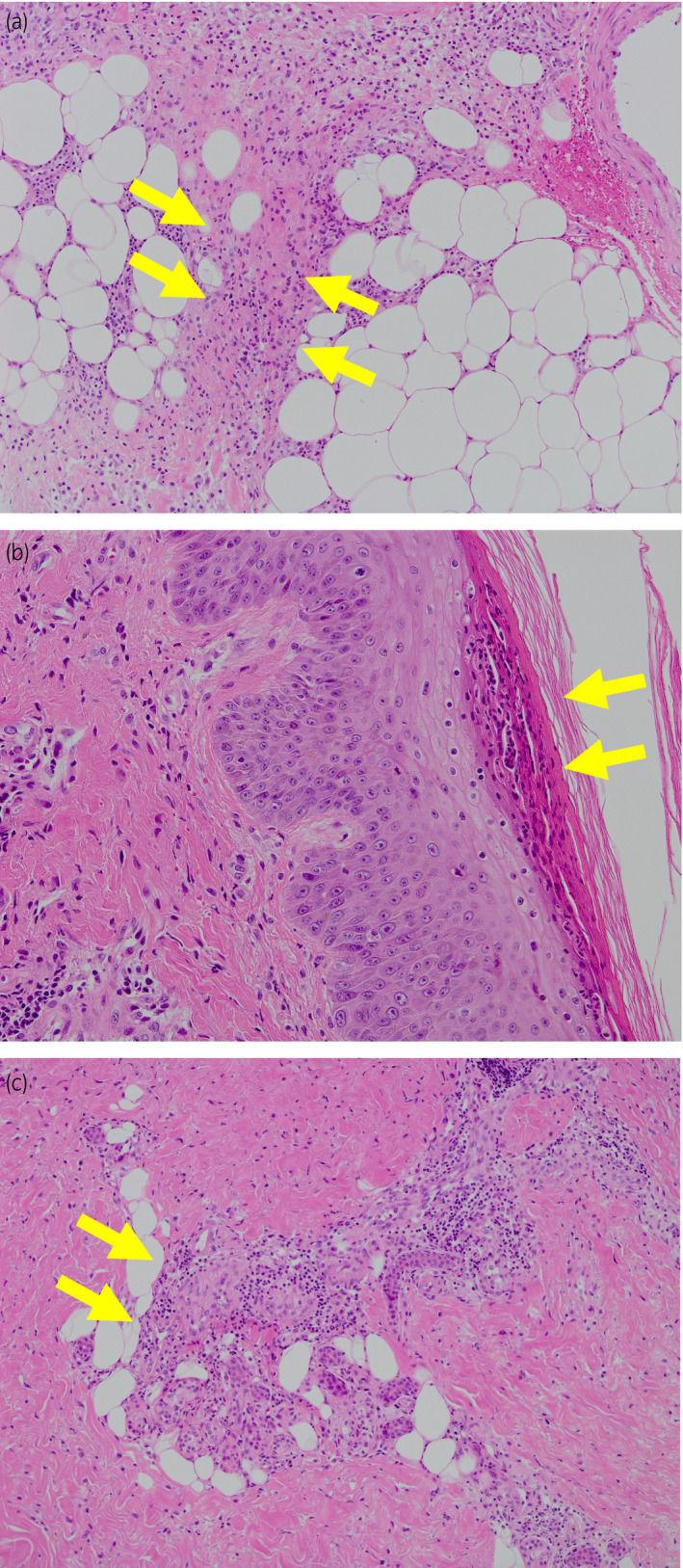
(a) Septal panniculitis (H&E staining). Yellow arrows indicate the septum of adipose tissue infiltrated with neutrophils and lymphocytes. (b) Pustule (H&E staining). Yellow arrows indicate neutrophil‐based pus accumulation in the fissures in the epidermis. (c) Perivascular lymphocytic and neutrophilic infiltration. Yellow arrows indicate infiltration of lymphocytes and neutrophils around vascular vessels.

**Fig. 3:**
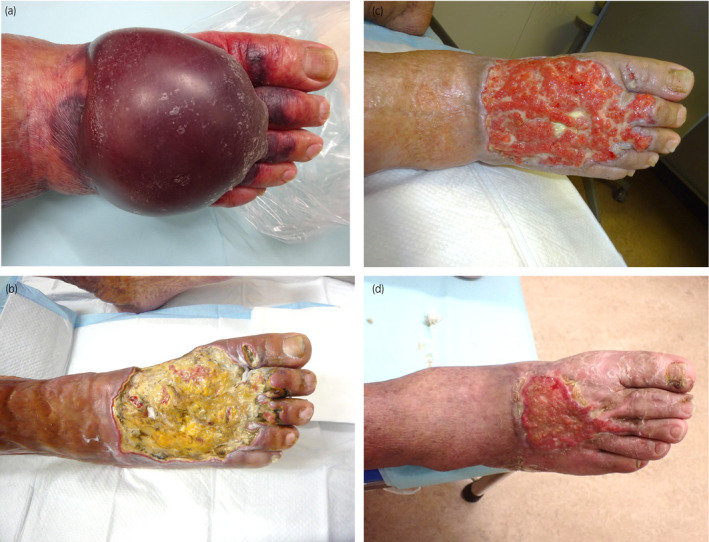
(a) Blister of the right dorsum of the foot. (b) Ulcer of the right dorsum of the foot before prednisolone treatment. (c) Six weeks after prednisolone treatment. (d) Seven months after prednisolone treatment.

A recently approved combination treatment with pembrolizumab (200 mg per 3 weeks) and axitinib (10 mg daily) was selected for metastatic RCC. One week after the first dose, edema of both lower legs developed (Fig. 1). At the end of the second course, axitinib was discontinued due to proteinuria (grade 3) and the development of hand‐foot syndrome (grade 2). At this point, CT scan showed mild shrinkage of the right renal tumor and multiple lung metastases. The sixth course of pembrolizumab was canceled due to loss of appetite and the patient was admitted to the hospital with urgent complaints of edema of the lower legs. At the time of admission, blisters appeared on the lower extremities and dorsum of the right foot. The blisters enlarged, and the beds of the blisters became ulcerous (Fig. 3a). Immediately after admission, skin biopsy was performed on the erythema of the right lower leg. Histopathological examination revealed septal panniculitis (Fig. 2a), pustules (Fig. 2b), and perivascular lymphocytic and neutrophilic infiltration (Fig. 2c) of the skin. Based on these various pathological findings and the progression of clinical findings, irAEs were strongly suspected. Oral prednisolone treatment (30 mg/day) was immediately started and gradually tapered as symptoms improved (Fig. 3b–d). Nine months after the onset, the skin lesion was smaller and almost epithelialized. As a second‐line treatment, the patient underwent cytoreductive right radical nephrectomy.

## Discussion

Immune checkpoint inhibition can cause diverse AEs in the organs, including the gastrointestinal tract, liver, heart, kidney, and skin. Cutaneous AEs occur approximately 5–9 weeks after the administration of PD‐1 or PD‐L1 inhibitors, which is earlier than most other AEs. Approximately 98% of patients who received combination therapy with pembrolizumab and axitinib experienced AEs. Among those, 14% of patients experienced dermatologic AEs.^10^ The incidences of dermatologic AEs during treatment with pembrolizumab, axitinib, and a combination of pembrolizumab and axitinib are summarized in Table 1. Hand‐foot syndrome occurred in 27% of axitinib‐treated patients and 28% of pembrolizumab plus axitinib‐treated patients. Rash was observed in both axitinib‐ and pembrolizumab‐treated patients. Blisters were only reported in patients treated with axitinib.^2^ Importantly, 1.9% and 1.2% of patients treated with a combination of pembrolizumab and axitinib experienced severe skin reactions of any grade and grades 3–4, respectively.^10^ Skin ulcers have been reported in 0.1% of patients treated with pembrolizumab^11^ and in 0.3% of patients undergoing axitinib treatment.^2^ Stevens‐Johnson syndrome and skin exfoliation have been reported as causes of skin ulcers due to pembrolizumab and axitinib treatment, respectively. There have been no reports of severe skin disorders in patients with preexisting stasis dermatitis.

**Table 1:** Incidence of skin‐related adverse events

	Any grade (%)	≧Grade 3 (%)
Rash		
Pembrolizumab	10.4	1.3
Axitinib	13	<1.0
Pembrolizumab/axitinib	14.2	0.2
Blister		
Pembrolizumab	NA	NA
Axitinib	1.1	0
Pembrolizumab/axitinib	NA	NA
Palmar‐plantar erythrodysesthesia syndrome		
Pembrolizumab	NA	NA
Axitinib	27	5
Pembrolizumab/axitinib	28	5.1
Severe skin reactions		
Pembrolizumab	5.2	5.2
Axitinib	NA	NA
Pembrolizumab/axitinib	1.9	1.2

Stasis dermatitis is an inflammation of the skin caused by chronic venous insufficiency in the lower legs. Symptoms include pruritus, scales, pigmentation, and sometimes ulcers. The diagnosis is clinically made.^12^ There have been no cases of stasis dermatitis caused by administration of pembrolizumab or axitinib reported in the past.

Immune‐related dermatitis presents with a variety of manifestations and pathological features such as superficial perivascular dermatitis, interface dermatitis, psoriasis, acantholytic dermatitis, acute generalized exanthematous pustulosis, and panniculitis. Erythema nodosum‐like panniculitis is a relatively rare dermatologic AE caused by immune checkpoint inhibitors.^13^, ^14^ In our case, septal panniculitis, pustules, and perivascular lymphocytic and neutrophilic infiltration were detected in the skin biopsy from erythema lesions, and a differential diagnosis of stasis dermatitis exacerbation and irAE was a subject of discussion. The first sign of immune‐related dermatitis at the end of the fifth course of pembrolizumab and axitinib was blisters in the lower extremities. Perivascular leakage of red blood cells and septal panniculitis on histopathological examination was a characteristic of stasis dermatitis. However, diverse findings, including lymphocytic and neutrophilic infiltration, and the progression of clinical findings led us to the diagnosis of immune‐related dermatitis. Although the relationship between stasis dermatitis and immune‐related panniculitis is unclear, it is possible that preexisting stasis dermatitis caused the onset and deterioration of immune‐related dermatitis in our case. In patients with preexisting stasis dermatitis, careful observation of skin finding is recommended during treatment with pembrolizumab and axitinib.

## Conclusion

We reported a case of immune‐related dermatitis in a patient with metastatic RCC treated with pembrolizumab and axitinib. Diverse pathological findings from skin biopsy led to a diagnosis of immune‐related dermatitis. Preexisting stasis dermatitis may have caused the onset and deterioration of immune‐related dermatitis. Similar case reports are currently limited.

## Conflict of interest

The authors declare no conflict of interest.

## Approval of the research protocol by an Institutional Reviewer Board

Not applicable.

## Informed consent

Written informed consent for publication was obtained from the patient.

## Registry and the registration no. of the study/trial

Not applicable.

## English language editing

The authors thank Editage (www.editage.com) for English language editing.

## Author contribution

Shunsuke Imai: writing – original draft. Masaki Nakamura: writing – original draft, writing‐review & editing, and supervision. Satomi Chujo: writing – review & editing. Ryousuke Ooki: writing – review & editing. Yasushi Inoue: writing – review & editing. Hajime Horiuchi: writing – review & editing. Teppei Morikawa: writing – review & editing. Keita Uchino: writing – review & editing. Atsuyuki Igarashi: writing – review & editing. Yoshiyuki Shiga: supervision.
